# Supplementary material for: Dysregulation of SIRT3 SUMOylation Confers AML Chemoresistance via Controlling HES1-Dependent Fatty Acid Oxidation
Source: Int J Mol Sci. 2022 Jul 27;23(15):8282. doi: 10.3390/ijms23158282 (PMC9368767; doi:10.3390/ijms23158282)
Supplement: Supplementary file 1 [file ijms-23-08282-s001.zip › ijms-1816160-supplementary.pdf]

PATIENT ID	SEX/AGE	STATUS	WHO CALSSIFICATION OF AML	MUTATIONS
AML02	M/31	De Novo	**AML, NOS**	None
AML03	F/69	De Novo	**AML, NOS**	None
AML11	M/40	Relapse	**AML, NOS**	TEL-ABL(+,108.97%)
AML12	M/54	Relapse	**AML-MRC**	None
AML17	F/41	De Novo	**AML, NOS**	RUNX1
AML18	M/54	Relapse	**AML, NOS**	None

Table S1. Clinical characteristics of AML patients. F: female; M: male. The table indicated 6 primary AML cells analyzed in our study, with their sex & age, disease stage and mutations listed in the corresponding rows.

Table S2: Primer sequences

Primers sequences for semi-quantitative PCR		
Name	Sense sequence (5'->3')	Antiense sequence (5'->3')
PCDH-SIRT3 WT	aatgaattcatggcgttctggggttggcgcgc	agccttaagctatttgtctggtccatcaagcttcc

Primer sequences for real-time PCR		
Name	Sense sequence (5'->3')	Antiense sequence (5'->3')
NFE2	taccgtgacattttccagca	aatcccatcagcagttccac
HES1	cacccctcctcctaaactcc	aggcgcaatccaatatgaac
LGR1	aaggcctgaatgggctaaat	tttgctgcatcagcagtacc
GAS6	cggaatctggtcatcaaggt	tcttctccgttcagccagtt
ID1	cggatctgagggagaacaag	tctgagaagcaccaaacgtg
CHI3L1	gatagcctccaacacccaga	aaattcggccttcatttcct
F3	gggctgacttcaatccatgt	gaaggtgcccagaataccaa
NRIP1	gcactgtggtcagactgcat	gtgttcacaagggcttggtt
BHLHE40	ccttgaagcatgtgaaagca	gcttggccagatactgaagc
G0S2	ccaaaggagtttgggattga	tgcaaaatggtggtcattgt

Figure S1

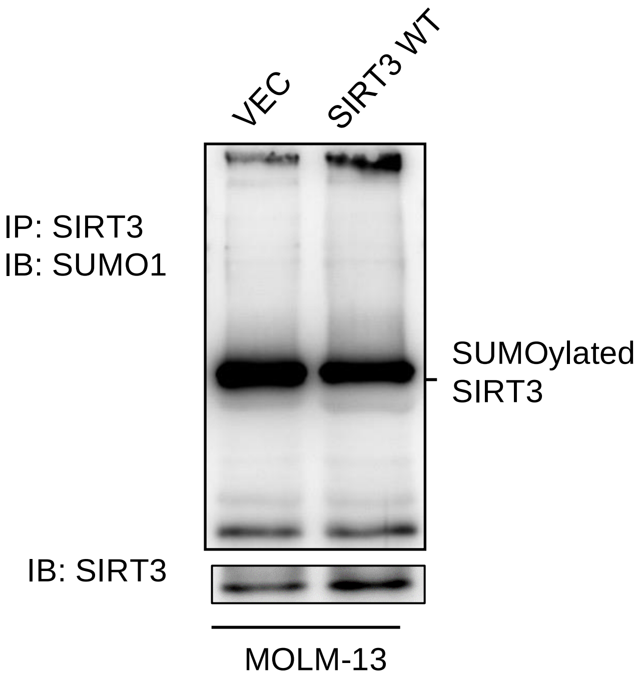

Figure S1: SIRT3 WT manifests higher level of SIRT3 SUMOylation in MOLM-13 cells. Whole cell lysates were isolated from lentiviral encoding vector control and SIRT3 WT overexpressing MOLM-13 cells before immunoprecipitated with anti-SIRT3 antibody followed by Immunoblotting with anti-SUMO1 antibody. 10% input protein was loaded to determine the expressions of endogenous SIRT3 and as an indication of equal loading.

Figure S2

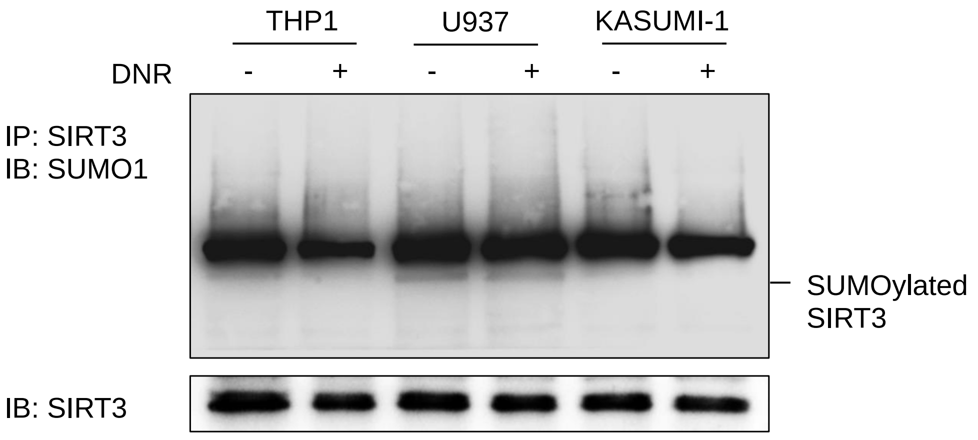

Figure S2: Chemotherapy attenuates SIRT3 SUMOylation in a panel of AML cell lines. Whole cell lysates were isolated from THP-1, U937 and KASUMI-1 cells that treated either with or without 50 nM DNR for 48 h prior to subject to immunoprecipitation with anti-SIRT3 antibody, followed by immunoblotting with anti-SUMO1 antibody. 10% input protein was loaded to determine the expressions of endogenous SIRT3 and as an indication of equal loading.

Figure S3

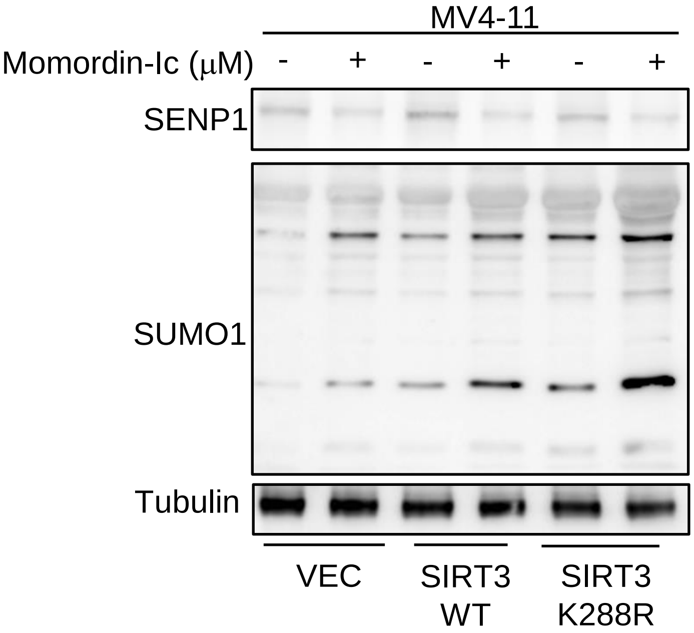

Figure S3: Momordin-Ic directly targeting on SENP1. Immunoblotting of SENP1, and SUMO1 protein expressions in either vector control, SIRT3 or SIRT3K288R overexpressing MV4-11 cells upon treatment with 25 mM momordin-Ic at 48 h time point. Lentiviral encoding vector control or SIRT3K288R transduced MV4-11 cells were treated either with or without 25 μM momordin-Ic for 48 h prior to treat with 300 μg/ml CHX at the indicated time points.

Figure S4

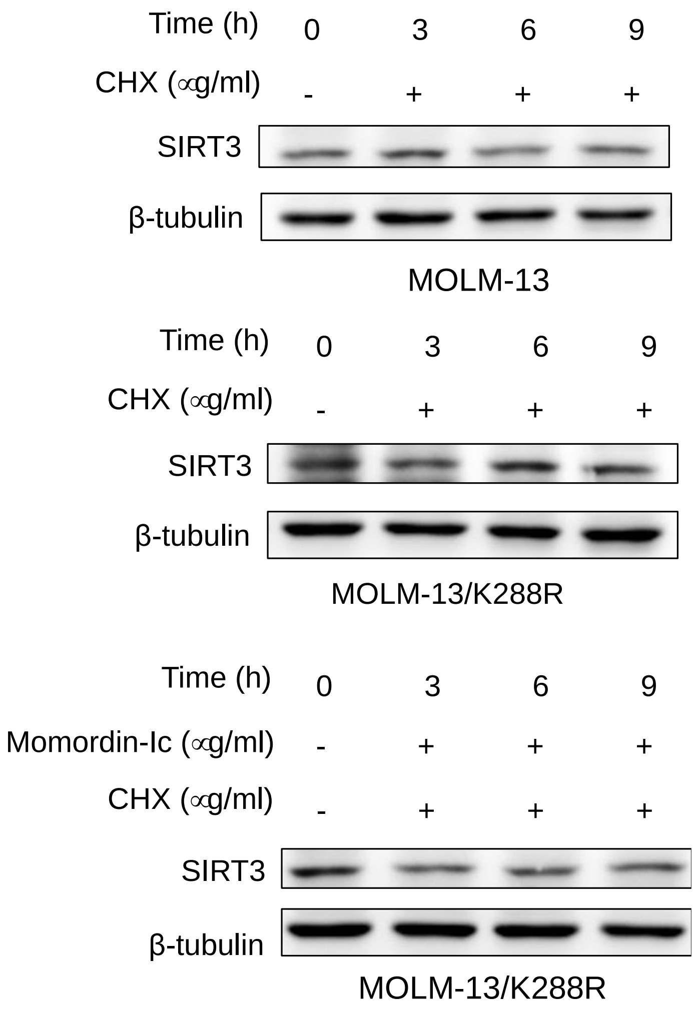

Figure S4: De-SUMOylation activates SIRT3 via inhibition of its protein degradation in MOLM-13 cells. Lentiviral encoding vector control or SIRT3K288R transduced MOLM-13 cells were treated either with or without 25 μM momordin-Ic for 48 h prior to treat with 300 μg/ml CHX at the indicated time points. β-tubulin was included as an indication of equal loading.

Figure S5

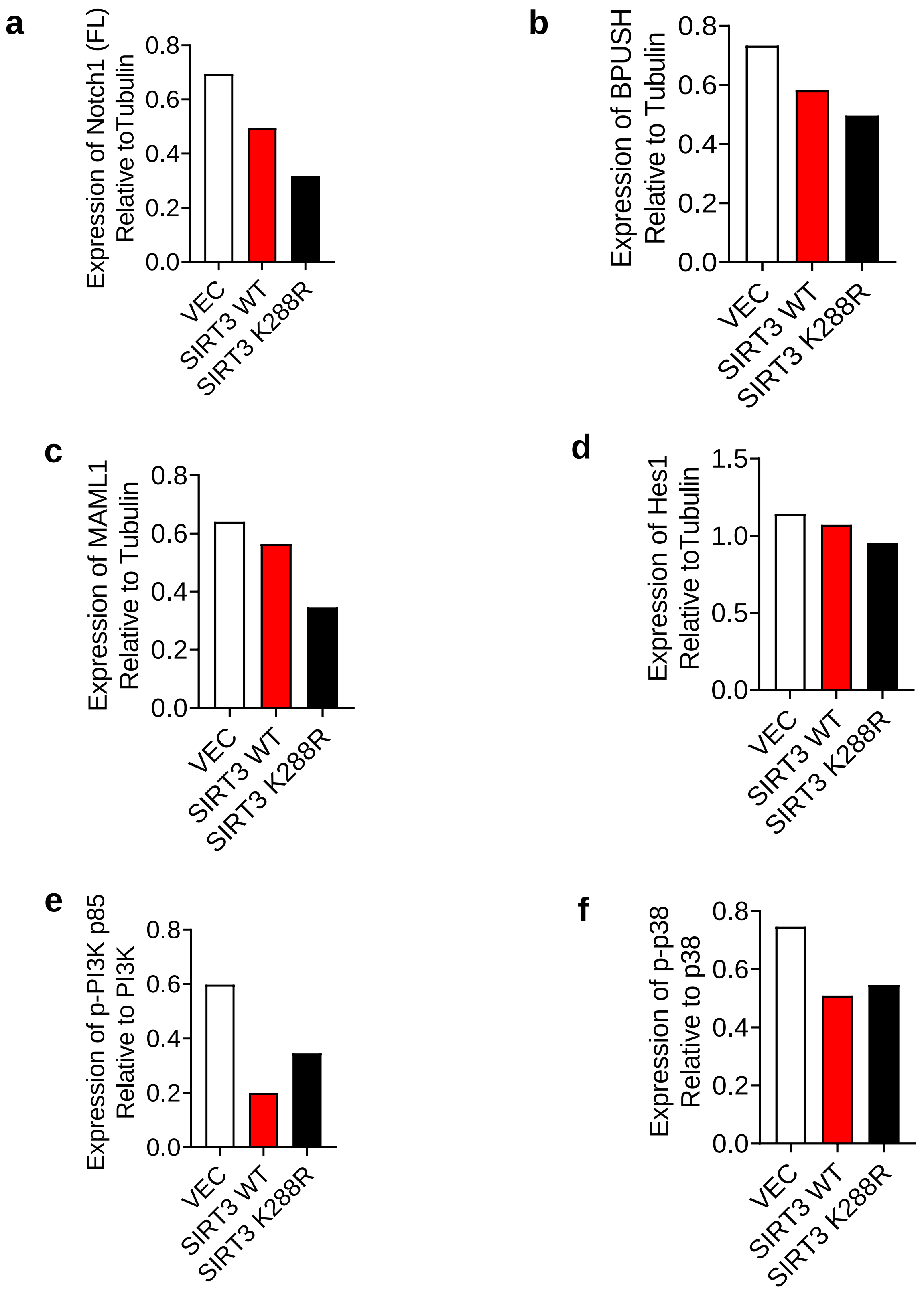

Figure S5: Densitometric quantification of Figure 5c. Immunoblotting assay of Notch 1, BPUSH, MAML1, HES1, and phosphorylated and total of PI3K, p38 proteins was performed as described previously. Bands intensities were measure using Image J software and the relative expressions of (a) Notch1 (b) BPUSH (c) MAML1 (d) Hes1 (e) p-PI3K (normalized to total PI3K) and (f) p-38 (normalized to total p38) were determined in lentiviral encoding vector control, SIRT3 and SIRT3K288R overexpressing MV4-11 cells.
